# Natural Killer T Cell Function in Lymphoma Patients

**DOI:** 10.3390/biom16050749

**Published:** 2026-05-20

**Authors:** Roshanak Derakhshandeh, Michael S. Lee, Yuyi Zhu, Emmanuel B. Asiedu, Jocelyn Reader, Rania H. Younis, Amy S. Kimball, Nicole Glynn, Michael Kallen, Tonya J. Webb

**Affiliations:** 1 Department of Microbiology and Immunology, University of Maryland School of Medicine, Baltimore, MD 21202, USA; roshanak.derakhshandeh@gunet.georgetown.edu (R.D.); yuyizhu1@som.umaryland.edu (Y.Z.);; 2 Marlene and Stewart Greenebaum Comprehensive Cancer Center, Baltimore, MD 21201, USArania.younis@gmail.com (R.H.Y.); kimball@amgen.com (A.S.K.); nglynn@umm.edu (N.G.); mkallen@som.umaryland.edu (M.K.); 3 Department of Obstetrics, Gynecology and Reproductive Sciences, University of Maryland School of Medicine, Baltimore, MD 21201, USA; 4 Department of Oncology and Diagnostic Sciences, School of Dentistry, University of Maryland, Baltimore, MD 21201, USA; 5 Department of Oncology and Diagnostic Sciences, School of Dentistry, Baltimore, MD 21201, USA; 6 Department of Pathology, University of Maryland School of Medicine, Baltimore, MD 21202, USA

**Keywords:** lymphoma, NKT cells, T-cells, lymphocytes, cytokines, cancer immunotherapy

## Abstract

Natural killer T (NKT) cells bridge innate and adaptive immune responses and play a critical role in anti-tumor immunity. The goal of the study was to assess NKT cell and T cell function in lymphoma patients and to investigate whether specific cytokines correlate with outcomes and/or immune cell function. Patient diagnoses were confirmed by histology. NKT and T cell number and function were assessed by flow cytometry and stimulation with artificial antigen-presenting cells (aAPCs) followed by ELISA and quantitative RT-PCR (qPCR). Cytokine expression levels were compared using online databases, and protein levels in the plasma were assessed by ELISA. NKT cell activation, indicated by at least 1.5-fold IFN-γ induction over baseline following stimulation, was detected in 82% of healthy donors, compared to 44% of lymphoma patients. Lymphoma patients have significantly higher levels of circulating pro- and anti-inflammatory cytokines IL-10, IL-6, and Sema4D as compared to healthy donors. In addition, NKT cell function in the blood correlated with NKT cell function in the bone marrow in lymphoma patients. We found that aAPC-qPCR can be used to quickly assess immune cell function in cancer patients. Circulating NKT cell function positively correlated with bone marrow NKT cell function, suggesting that circulating NKT responses reflect systemic immune competence. Outcome-associated transcriptomic analyses showed that lower expression of TGF-β, IL-6, IL-10, and IFN-γ mRNA correlated with poorer clinical outcomes, whereas higher Sema4D expression was associated with worse prognosis, identifying Sema4D as a potential immunologic biomarker linked to disease progression and immune dysfunction in B cell lymphoma.

## 1. Introduction

Natural killer T (NKT) cells are a unique subset of lymphocytes that activate and bridge the innate and adaptive immune systems [1]. Unlike classic CD4 and CD8 T cells, NKT cells are evolutionarily conserved. The majority of NKT cells express an invariant TCRα chain, called Vα24Jα18 in humans and associated with Vβ11 [2,3,4,5]. NKT cells recognize glycolipid antigens within the context of CD1d, a non-classical MHC class I-like molecule [6]. NKT cells have the capacity to mount strong anti-tumor responses and are a major focus in the development of effective cancer immunotherapy [7,8]. NKT cells have been shown to augment anti-tumor responses due to their rapid production of large amounts of IFN-γ after stimulation, which helps to activate other immune cells. We and others have generated strong evidence, in a variety of mouse-based tumor models, that treatment with α-GalCer, a specific and potent activator of NKT cells, can lead to tumor eradication [9,10,11,12]. Given the potentially high therapeutic significance of NKT cell-based therapies, clinical trials have been conducted in cancer patients, and there have been no reports of overt toxicity associated with NKT cell-based immunotherapy to date [13,14]. However, one limitation of translating this biology in therapy has been the low number of circulating NKT cells in the blood and a further reduction often found in cancer patients. To address this potential limitation, we developed methods that can be used to expand NKT cells from blood [15], as well as from human stem progenitor cells (HPSC) [16], to test the preclinical efficacy of adoptive immunotherapy, such as chimeric antigen receptor (CAR)-based approaches [17].

While the use of chimeric antigen receptors (CARs) has revolutionized the field of adoptive immunotherapy, particularly in patients with B-cell malignancies [18,19,20,21], recent interest in using an NKT cell carrier rather than T cells has gained traction, because CD1d is relatively monomorphic and has a reduced risk of mediating graft-versus-host disease (GVHD) [22,23,24,25,26]. Preclinical studies have shown that hematopoietic stem cell-NKT cell-based therapy can be effective for the treatment of hematologic malignancies [22]. Besides tumor regression, studies have shown that CAR-GD2 NKT cells eliminate immunosuppressive CD1d-positive M2 macrophages in vitro [27]. Rotolo et al. optimized CAR-NKT cell production and showed the benefit of a single dose of CAR19-iNKT over CAR-T cells in terms of both tumor-free and overall survival in a mouse model of CD1d+CD19+ B lineage cancer, without resulting in GVHD [28]. In addition, studies by Yang and colleagues have shown that allogeneic CAR-NKT cells can effectively target multiple cancer types, and their studies highlight the antitumor efficacy, expansion and persistence of stem cell-derived allogeneic CAR-NKTs [29,30]. These studies suggest that NKT cells have greater potential for the treatment of cancer compared to conventional T cells, as their activation can induce both the innate and adaptive arms of the immune system and result in long-lasting protection.

However, it is currently unclear which patients would benefit from NKT cell-based immunotherapy. We recently examined NKT cell number and function in breast cancer patients and identified unique immune gene signatures in patients with functional NKT cells [31]. Therefore, we hypothesized that an assessment of the stimulatory capacity of circulating NKT/T cells and cytokine levels in patients with B cell malignancies could facilitate improvements in the diagnostic workup to identify personalized treatment options for lymphoma patients. In the current study, we assessed total T cell and NKT cell function in lymphoma patients compared to healthy donors using an artificial antigen-presenting cell (aAPC) stimulation protocol in combination with quantitative RT-PCR (qPCR) [15]. Our assay was used to assess IFN-γ induction as a marker of NKT/T cell activation. In addition, we measured several cytokines in the plasma of lymphoma patients to determine if levels correlated with immune cell function or relapse. We found that TGF-β1 levels inversely correlated with relapse within our cohort. Therefore, the development of immune profiles using NKT cell function along with plasma cytokines levels may help to define the immune status of patients and may one day contribute to treatment decisions.

## 2. Materials and Methods

### 2.1. Study Design

Peripheral blood was collected from lymphoma patients at the University of Maryland Greenbaum Cancer Center prior to treatment. Written informed consent was obtained from all lymphoma patients before participation, and the Institutional Review Board of the University of Maryland School of Medicine approved this study (protocol GCC 1367, approval date: 19 August 2025). This study included 34 healthy donors and 54 lymphoma patients diagnosed with outcomes available for 44 lymphoma patients (relapse: *n* = 18; no relapse: *n* = 26), with a median follow-up of 60 months from diagnosis. Samples from 50 patients were analyzed in the initial studies and are included in Table 1. In addition, lymphoma patients (*n* = 5) were utilized in a follow-up experiment in which we compared the function of NKT cells in the blood and bone marrow (BM) from the same patient, including four patients not included in the original analysis. Forty-one of the lymphoma patients were newly diagnosed, and specimens were obtained prior to treatment. Residual blood specimens obtained from lymphoma patients that were candidates for stem cell transplantation were also used for this study *(n* = 13). When the post-treatment outcomes were available, they were used to categorize patients into those who did or did not experience relapse for subsequent comparisons.

### 2.2. Isolation of Human PBMCs and Plasma

Lymphoma patient peripheral blood mononuclear cells (PBMCs) and bone marrow cells were isolated using BD Vacutainer CPT Tubes containing sodium heparin anticoagulant (362753; Fisher Scientific, Suwanee, GA, USA) (*n* = 41). PBMCs were also collected from cryopreservation bags (*n* = 13). Healthy donor PBMCs were isolated from CPT tubes or buffy coats (purchased from BioIVT, Woodbury, NY, USA) using Ficoll-Hypaque (Amersham Pharmacia Biotek, Uppsala, Sweden) in SepMate-50 tubes (StemCell Technologies, Vancouver, BC, Canada) diluted in 2% FBS in PBS, according to the manufacturer’s instructions. Plasma was obtained from the top fraction of the CPT tubes.

### 2.3. Generation of aAPC

CD1d-aAPCs and anti-CD3/CD28 beads were generated as previously described [32]. Briefly, hCD1d-Ig (recombinant soluble dimeric Human CD1d:Ig Fusion Protein; BD Pharmingen, San Jose, CA, USA; catalog No. 557764) or anti-CD3 mAb (Biolegend, San Diego, CA, USA; catalog No. 317303) and anti-CD28 mAb (Biolegend; catalog No. 302933) were conjugated to M-450 Dynabeads (ThermoFisher, Waltham, MA, USA; catalog No. 14011). The CD1d-aAPCs were then loaded with α-Galactosylceramide (Axxora, London UK, catalog No. BV-2152-1000).

### 2.4. Stimulation of PBMCs and Bone Marrow Cells

One million PBMCs were stimulated for 4 h at 37 °C, with an equal number of anti-CD3-CD28-coated beads or α-GalCer-loaded CD1d-aAPCs in complete media containing RPMI 1640 Medium, non-essential amino acids [Sigma-Aldrich, St. Louis, MO, USA], sodium pyruvate [Gibco, Invitrogen Corporation, Waltham, MA, USA], vitamin solution [Gibco], 2-mercaptoethanol [Gibco], and 10 μM ciprofloxacin [Serologicals Proteins Inc, Norcross, GA, USA] and supplemented with 5% human AB serum. The cell culture medium/empty beads or unloaded aAPCs served as negative controls. PMA (50 ng/mL) and ionomycin (1 μM) were used as a positive control. NKT and T cell function was calculated as the relative induction of IFN-γ (as described in the next section) by α-GalCer-loaded CD1d-aAPC or anti-CD3-CD28-coated beads, respectively, over the induction of IFN-γ by the negative control. In follow-up studies comparing PBMC and BM samples, cell function is set relative to fold induction of the positive control to generate a “percent max” in order to account for differences in sample quality after extended storage.

### 2.5. Real-Time Quantitative PCR (qPCR)

RNA was isolated using the RNeasy Plus Kit (Qiagen, Germantown, MD, USA) according to the manufacturer’s protocol. RNA concentration and purity were determined using the Take 3 plate and the Synergy H1 Hybrid reader. Reverse transcription PCR was performed using the iScript cDNA Synthesis Kit (Biorad, Hercules, CA, USA) according to the manufacturer’s directions. To measure the induction of IFN-γ mRNA in the stimulated PBMC, qPCR was performed. The ABI Sybr Green master mix and HotStart Taq master mix kit from QIAGEN were used. qPCR was performed using proprietary sequences generated by Qiagen that were specific for IFN-γ (cat. #PPH00380C) and 18S (cat. #PPH05666E) [31]. The total volume of the reaction mix was 20 μL and consisted of 10 μL master mix, 1 μL primer mix (3 μM), 5 μL H2O, and 4 μL cDNA (diluted 1:10). The Applied Biosystems 7500 Fast Real Time PCR system was used. The CT values were collected, and the fold increase calculated as follows: n-fold increase in IFN-γ mRNA = 2[−(CT_sample_ − CT 18S rRNA) − (CT_empty beads_ − CT 18S rRNA)], where CT is the threshold cycle.

### 2.6. ELISA

IL-10 and IL-6 levels from healthy donor or lymphoma patient plasma were determined by standard sandwich ELISA (BioLegend, catalog No. 430607 and 430507). Semaphorin 4D (Sema4D) concentration in the plasma was determined using direct ELISA. In brief, Immulon 4 HBX microtiter plates (Thermo Scientific, Waltham, MA, USA) were coated with 50 microliters of undiluted plasma, washed, then incubated with anti-human CD100 antibody (clone: 133-1C6; Novus Biologicals, Centennial, CO, USA). Goat anti-mouse IgM-HRP (catalog No. M31507; Life Technologies, Carlsbad, CA, USA) was added, followed by detection with TMB (Pierce, Appleton, WI, USA). The plasma concentrations of Sema4D were calculated using the standard curve established for recombinant Sema4D (catalog No. 310-29; Peprotech, Rocky Hill, NJ, USA). The detection limit was 3.1 ng/mL. Mouse IgM isotype control was used for the direct ELISA assay (Abcam, catalog No. ab91546). For detection of TGF-β1, the Human ELISA TGF-β1 total kit and Free Active TGF-β1 kit were used following the manufacturer’s recommendations, using patient or healthy donor plasma (catalog No. 436707; BioLegend, San Diego, CA, USA). IFN-γ levels following stimulation of PBMC were determined by standard sandwich ELISA (Biolegend). All samples were run in triplicate, and plates were read using a BioTek Synergy microplate spectrophotometer at a 450 nm wavelength.

### 2.7. Statistical Analysis

Study data were plotted using box and whisker, bar, or scatter plots, using the mean +/− SEM. Distribution of the continuous variables was compared between two groups using the two-tailed *t*-test with Welch’s correction. Linear regression was used for correlation analysis between PBMCs and BM samples. All statistical analyses were performed using Prism v11 software (GraphPad), and the details are included in the figure legends.

## 3. Results

### 3.1. NKT Cell Function in Lymphoma Patients

**Figure 1:**
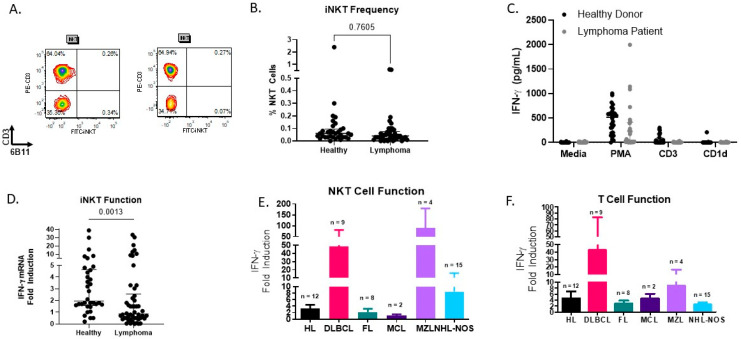
Number and function of circulating NKT cells from lymphoma patients and healthy donors. (**A**) Peripheral blood mononuclear cells (PBMCs) were isolated from healthy donors and cancer patients. Cells were stained for Va24+Vb11+ or iNKT+(6B11) CD3+ and analyzed by flow cytometry. Data from two healthy donors are shown. (**B**) Scatterplots demonstrate the variation in the percentages of NKT cells. Healthy donors *n* = 33; lymphoma patients *n* = 45. (**C**) PBMC from healthy donors and lymphoma patients were incubated for 4 h with media PMA/ionomycin (PMA), anti-CD3/CD28 (CD3) microbeads, or CD1d-aAPC (CD1d), and NKT/T cell activation was assessed by measuring IFN-γ levels by ELISA. (**D**) Circulating NKT cells from lymphoma patients are functionally impaired. PBMC were stimulated with CD1D-Ig/aCD28 aAPC loaded with α-GalCer to activate NKT cells or anti-CD3/CD28 microbeads to stimulate T cells for 4 h. RNA was extracted and qPCR was performed to assess IFN-γ and 18S. Fold induction was calculated relative to the control (cells stimulated with empty beads). Healthy donors *n* = 34; lymphoma patients *n* = 50. (**E**) NKT cell function in specific lymphoma subtypes. (**F**) Total T cell function in specific lymphoma subtypes. HL: Hodgkin’s lymphoma; NHL-NOS: non-Hodgkin’s lymphoma—not otherwise specified; DLBCL: diffuse large B cell lymphoma; FL: follicular lymphoma; MCL: mantle cell lymphoma; MZL: marginal zone lymphoma.

We first measured the percentage of NKT cells in the peripheral blood mononuclear cell (PBMC) fraction of healthy donors and lymphoma patients (Figure 1A). The percentage of NKT cells in both healthy donors and lymphoma patients prior to treatment was relatively low. We found that the percentage of NKT cells was slightly, but not significantly, lower in lymphoma patient PBMCs (0.07 ± 0.02) compared to healthy donors (0.14 ± 0.07) (Figure 1B). This decrease in NKT cell percentage in lymphoma patient PBMCs is consistent with previously published studies.

As we observed a low percentage of circulating NKT cells, we next sought to determine if stimulation with artificial antigen-presenting cells (aAPCs) could be used to assess NKT cell function in lymphoma patients. PBMCs were stimulated with α-GalCer-loaded aAPC, and IFN-γ induction was measured by ELISA (Figure 1C). Similar to our previous studies, we found that it was rare to detect IFN-γ production by NKT cells following a four-hour activation period. Next, we sought to measure both total T and NKT cell function in healthy donors and lymphoma patients using anti-CD3-CD28-coated beads or our previously established aAPC-qPCR method (Table 1; patient data shown in Supplemental Figure S1). We found that total T cell function trended downward in lymphoma patients relative to healthy donors (5.136 ± 1.61 vs. 22.540 ± 13.07 IFN-γ induction fold change) (Table 1). It has been previously reported that, upon stimulation with α-GalCer, NKT cells from mice with lymphoma produce less IFN-γ than NKT cells from healthy mice [12]. Similarly, NKT cells from healthy donors showed a greater level of activation, as measured by fold induction of IFN-γ, compared to NKT cells from lymphoma patients (4.97 ± 1.39 vs. 3.72 ± 1.02), although this difference failed to reach statistical significance (Figure 1D). In addition, we were only able to detect NKT cell function, as determined by a greater than 1.5-fold increase in IFN-γ, in 44% of our lymphoma patients (*n* = 22/50) compared to 82% of our healthy donors (*n* = 28/34).

After detecting a decrease in both NKT cell number and function in lymphoma patients compared to healthy donors, we further investigated our cohort of lymphoma patients. While total T cell function and NKT cell function were similar for the majority of patients, there are instances, such as in MZL, where NKT and T cell trends are different, as shown in Figure 1E,F. Additionally, the fact that the MCL patients had the lowest level of NKT cell function is interesting and aligns with our prior report, which found that NKT cells from human MCL patients failed to expand to the same extent as healthy donor NKT cells when cultured with aAPCs or to produce IFN-γ when stimulated with anti-CD3-CD28 beads or PMA and ionomycin [16]. Thus, our data suggest that NKT cell function is a unique marker, distinct from T cell function.

### 3.2. Study Participant Demographics

This study included 34 healthy donors and 54 lymphoma patients. Among the lymphoma patients, outcomes were available for 44 patients, including 18 patients who experienced a relapse and 26 patients who did not experience a relapse. Table 1 shows the characteristics of both healthy donors and lymphoma patients. Our study includes patients with a range of lymphoma subtypes, including both Hodgkin’s lymphoma (HL; 24%), and non-Hodgkin’s lymphoma (NHL; 76%). Our NHL patients included mantle cell lymphoma (MCL; 4%), marginal zone lymphoma (MZL; 8%), follicular lymphoma (FL; 18%), diffuse large B cell lymphoma (DLBCL; 20%), and not otherwise specified (NHL-NOS; 26%).

**Figure 2:**
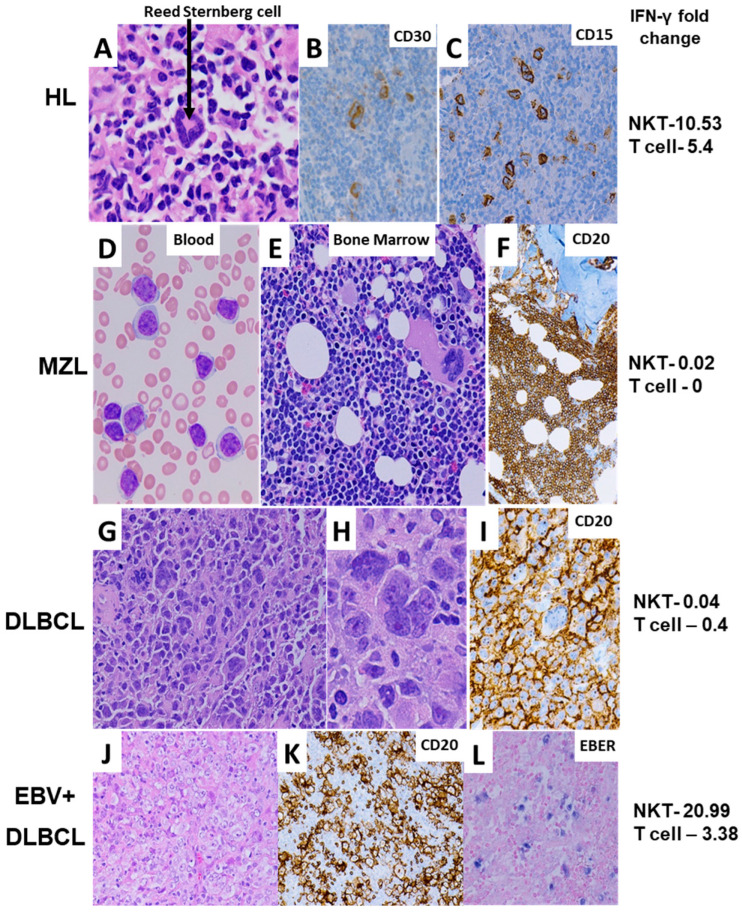
Histologic images. (**A**–**C**) Classic Hodgkin’s lymphoma, demonstrating a mixed inflammatory background and a neoplastic large binucleated Reed Sternberg cell ((**A**–**H**&**E**), 1000×). These are immunoreactive for CD30 ((**B**)—400×) and CD15 ((**C**)—400×). (**D**–**F**) Marginal zone lymphoma. A peripheral blood smear ((**D**)—Wright Giemsa stain, 1000×) demonstrates atypical lymphocytosis comprised of medium-sized cells with mature chromatin and moderate amounts of pale cytoplasm. The bone marrow ((**E**—**H**&**E**), 400×) was diffusely involved by a mature atypical lymphoid proliferation, forming interstitial sheets, which was strongly CD20-positive ((**F**)—200×), and the CD5-/CD10-/CD103-/kappa light chain was restricted by flow cytometry. (**G**–**I**) Diffuse large B-cell lymphoma, anaplastic variant, featuring sheets of large highly irregular cells ((**G**—**H**&**E**), 400×) with marked anaplasia ((**H**—**H**&**E**), 1000×) and diffuse CD20 immunoreactivity ((**I**)—400×). (**J**–**L**) EBV-positive diffuse large B-cell lymphoma, formerly of the elderly, displays ((**J**–**H**&**E**), 400×) sheets of large, atypical lymphocytes with irregular nuclear contours, vesicular chromatin, and prominent nucleoli, with ((**K**)—400×) diffuse strong CD20 staining highlighting large irregular forms and ((**L**)—1000×) EBER positivity as indicated by in situ hybridization staining.

The patients’ diagnoses were confirmed by histology. Figure 2 shows representative histologic images of patients in this study, highlighting key features supporting the diagnoses attributed to these patients. These representative patients include HL (Figure 2A–C, NKT cell function = 10.53, T cell function = 5.4, relapse), MZL (Figure 2D–F, NKT cell function = 0.02, T cell function = 0, relapse), anaplastic variant DLBCL (Figure 2G–I, NKT cell function = 0.04, T cell function = 0.4, no relapse), and EBV-positive DLBCL (Figure 2J–L, NKT cell function = 20.99, T cell function = 3.38, no relapse). In addition, the distribution of females and males included in this study was similar for both healthy donors (52.9% female) and lymphoma patients (46.9% female).

It is worth noting that, while not the focus of this study, we observed differences in NKT cell function between female (2.54 ± 0.6) and male (7.7 ± 2.81) healthy donors (Table 1). This decrease in NKT cell function, as determined by fold induction of IFN-γ, in females relative to males has been previously reported [33]. In addition to including a range of lymphoma subtypes, this study also included lymphoma patients with diverse ages and racial backgrounds. While 44% of the patients with age information available were in the 60+ age range, compared to 11% in the healthy donor group (Table 1), the youngest patient included was 25 years old, while the oldest was 72 years old. Finally, this study included patients from multiple racial backgrounds, including Black (15.6%), Hispanic (6.3%), Asian (3.1%), and White (75%), among those for whom racial background information is available.

### 3.3. Cytokine Levels Are Altered in Lymphoma Patients

**Figure 3:**
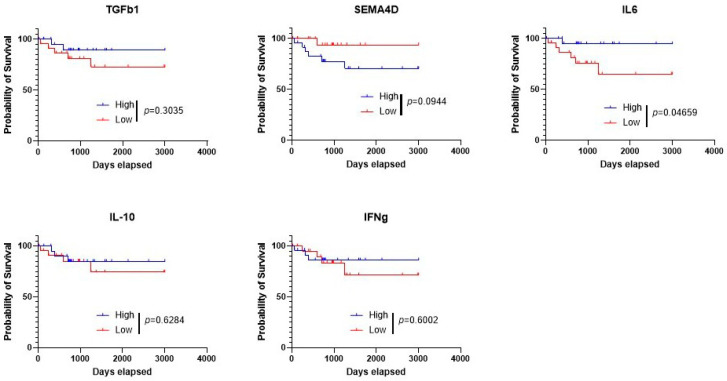
Lower mRNA Levels of TGF-β, IL-6, IL-10, and IFN-γ are correlated with poor outcomes in lymphoma patients. Patients were stratified based on high or low expression of selected genes based on mRNA expression. Time until death, overall survival days elapsed, and mRNA expression levels was assessed. The data were obtained from UCSD Xena. The dataset was from the GDC TGCA Large B Cell Lymphoma cohort. A log-rank (Mantel–Cox) test was used to determine if the survival curves are significantly different, with the *p* value indicated above.

NKT cells are known to produce a wide variety of both pro- and anti-inflammatory cytokines, and we speculated that an assessment of cytokines would both provide insight into the mechanism of NKT cell dysfunction in lymphoma patients and serve as a prognostic factor for patients. Notably, cytokines are derived from a variety of cellular sources and are not specific to NKT cells. We focused on five cytokines: IFN-γ (pro-inflammatory), IL-10 (anti-inflammatory), IL-6 (pro-inflammatory), Semaphorin 4D (SEMA4D; anti-inflammatory), and TGF-β1 (anti-inflammatory). We found that lower levels of TGF-β, IL-6, IL-10, and IFN-γ mRNA were associated with poorer outcomes, in contrast to Sema4D, in which higher levels correlated with worse outcomes (Figure 3).

**Figure 4:**
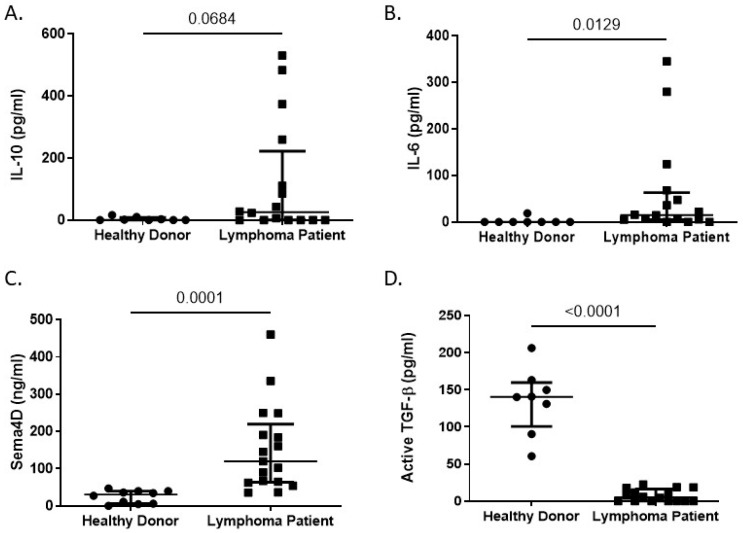
Cytokine levels in healthy donors and lymphoma patients. Plasma from healthy donors (*n* = 8) or lymphoma patients (*n* = 16) was analyzed by ELISA for levels of (**A**) IL-10, (**B**) IL-6, (**C**) Sema4D, and (**D**) TGF-β. For each panel (**A**–**D**), a Shapiro–Wilk test and an F test of variances failed to demonstrate normality of the residuals (*p* < 0.0001) and homoscedasticity (*p* < 0.0001), respectively. Thus, a nonparametric, Kolmogorov–Smirnov test was performed, with the *p*-value included above.

When we assessed plasma cytokine levels in our patient cohort, we found that lymphoma patients had significantly elevated levels of IL-10 (116.3 ± 46.97 vs. 2.190 ± 2.76 pg/mL), IL-6 (60.66 ± 26.08 vs. 0 pg/mL) and Sema4D (153.3 ± 28.24 vs. 24.59 ± 5.5 ng/mL) compared to healthy donors (Figure 4A–C). Of all the elevated cytokines, Sema4D levels were the most significantly upregulated (*p* = 0.002). Sema4D was recently implicated as a biomarker in human head and neck squamous cell carcinoma, with higher levels found in those with disease compared to healthy donors [34]. The data in this study may justify expanding the range of cancers in which Sema4D can serve as a biomarker.

We also found that lymphoma patients had significantly decreased levels of TGF-β1 compared to healthy donors (6.23 ± 2.37 vs. 135.2 ± 15.63 pg/mL) (Figure 4D). TGF-β1 is commonly viewed as an immunosuppressive cytokine and has been shown to be produced by immunosuppressive cells, such as myeloid-derived suppressor cells, to suppress anti-tumor immunity and promote tumor growth [35]. In addition, expression of TGF-β1 mRNA was found to be twice as high in patients diagnosed with high-grade lymphomas compared to patients with low-grade lymphomas [36]. Therefore, our data were unexpected, as TGF-β1 was found to be significantly decreased in cancer patients compared to healthy donors.

### 3.4. TGF-β1 Levels Correlate with Relapse in Lymphoma Patients

**Figure 5:**
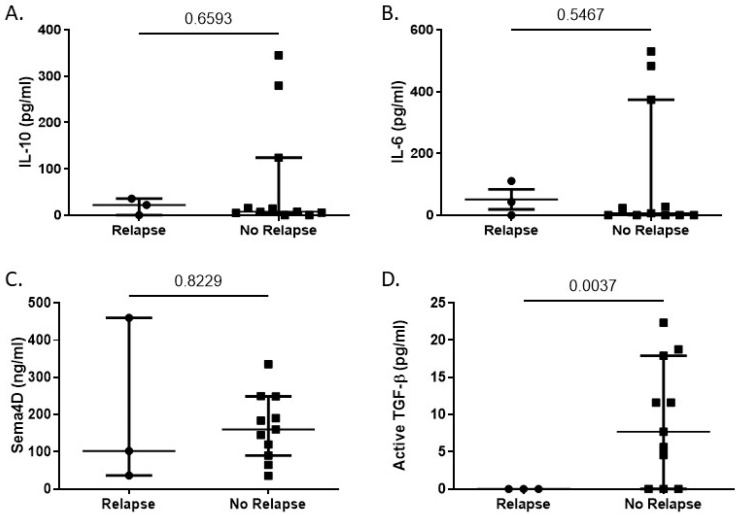
TGF-β levels inversely correlate with relapse in lymphoma patients. Plasma from lymphoma patients who either experienced relapse (*n* = 3) or did not experience relapse (*n* = 11) was analyzed by ELISA for levels of (**A**) IL-10, (**B**) IL-6, (**C**) Sema4D, and (**D**) TGF-β1. Statistical analyses for each panel were performed as indicated: (**A**) Shapiro–Wilk test and an F test of variances failed to demonstrate normality of the residuals (*p* = 0.0004) and homoscedasticity (*p* = 0.0417), respectively. Thus, a nonparametric, Kolmogorov–Smirnov test was performed. (**B**) A Shapiro–Wilk test failed to demonstrate normality of the residuals (*p* = 0.0007). Thus, a nonparametric, Kolmogorov–Smirnov test was performed. (**C**) A Shapiro–Wilk test demonstrated normality of the residuals (*p* = 0.5874). Thus, a two-tailed Welch *t* Test was performed. (**D**) A Shapiro–Wilk test demonstrated normality of the residuals (*p* = 0.3359). Thus, a two-tailed Welch *t* Test was performed, with the *p*-value included above.

After determining differences in the cytokine profiles of lymphoma patients compared to healthy donors, we focused on the possibility that cytokine levels could serve as a predictive factor for relapse within our lymphoma patient cohort. After excluding patients that did not have outcome data available (*n* = 6), we examined the cytokine levels in lymphoma patients who did or did not experience relapse after therapy at the time of this study (*n* = 44). Of those 44 patients, 14 had a sufficient sample quantity available to examine cytokine levels in addition to NKT and T cell function. We found no correlation between relapse and the levels of IL-10, IL-6 or Sema4D at diagnosis (Figure 5A–C). We found that, with a median follow-up of 60 months, lymphoma patients who experienced a relapse had significantly lower levels of TGF-β prior to treatment compared to those who did not experience a relapse (0 vs. 9.1 ± 2.42 pg/mL) (Figure 5D). This was once again unexpected, as high levels of TGF-β are associated with immunosuppression and have been implicated in promoting tumor growth [35,36].

### 3.5. NKT Cell Function Is a Predictive Factor for Relapse in Lymphoma Patients

**Figure 6:**
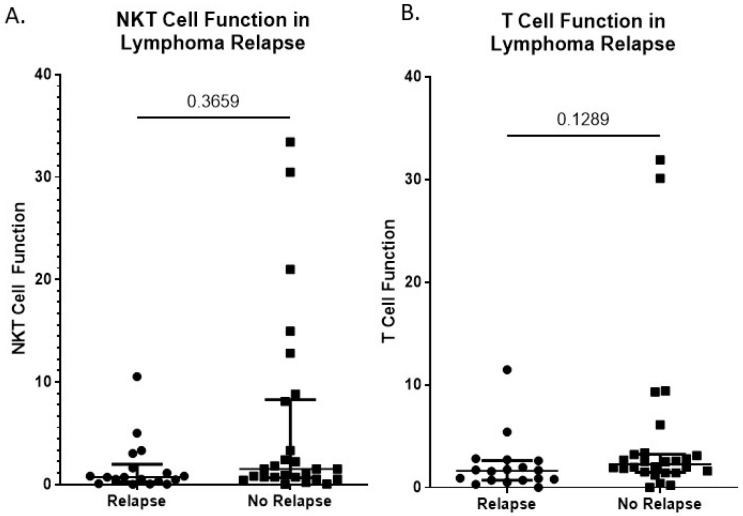
Reduced NKT cell and T cell function in relapsed lymphoma patients. PBMCs were stimulated with CD1D-Ig/aCD28 aAPC loaded with α-GalCer to activate NKT cells or anti-CD3/CD28 microbeads to stimulate T cells for 4 h. RNA was extracted and qPCR was performed to assess IFN-g and 18S. Fold induction was calculated relative to the control (cells stimulated with empty beads). (**A**) NKT cell and (**B**) T cell function in lymphoma patients who did (*n* = 18) or did not (*n* = 26) relapse. Statistical analyses for each panel were performed as indicated: (**A**) A Shapiro–Wilk test and an F test of variances failed to demonstrate normality of the residuals (*p* < 0.0001) and homoscedasticity (*p* < 0.0001), respectively. Thus, a nonparametric, Kolmogorov–Smirnov test was performed, with the *p*-value included above. (**B**) A Shapiro–Wilk test and an F test of variances failed to demonstrate normality of the residuals (*p* < 0.0001) and homoscedasticity (*p* < 0.0001), respectively. Thus, a nonparametric, Kolmogorov–Smirnov test was performed, with the *p*-value included above.

We previously identified NKT function as an independent marker of T cell function, which detected activation in 82% of healthy donors (*n* = 28/34) compared to 44% of lymphoma patients (*n* = 22/50) (Figure 2D). Therefore, we sought to determine whether our aAPC-qPCR platform for assessing NKT cell function could be used as a predictive factor for relapse in lymphoma patients. Patients in our lymphoma cohort who did not have outcome data available were excluded, and the 44 remaining were categorized into those that either did or did not experience relapse. There was no significant difference in the percentage of NKT cells between lymphoma patients who did or did not (0.04 ± 0.04 vs. 0.10 ± 0.15% NKT cells) experience a relapse (Figure S2A). This held true when examining specific lymphoma subtypes as well (Figure S2B–F). We found that NKT cell function trended higher in patients who did not experience a relapse compared to those who did (5.78 ± 1.83 vs. 1.64 ± 0.61 IFN-γ induction fold change) (Figure 5A). However, further analysis did not reveal any specific lymphoma subtype with a significant difference in NKT cell function between patients who did and did not relapse, which may be due to limited patient numbers, although the difference appeared to be greatest for diffuse large B cell and follicular lymphoma patients (Figure S3A–E). T cell function, as measured, did not show significant differences between lymphoma patients who did or did not (2.13 ± 0.62 vs. 4.89 ± 1.57 IFN-γ induction fold change) experience relapse (Figure 6B). Further analysis did not reveal any specific lymphoma subtype with a significant difference in T cell function between patients who did and did not relapse (Figure S3F–J).

**Figure 7:**
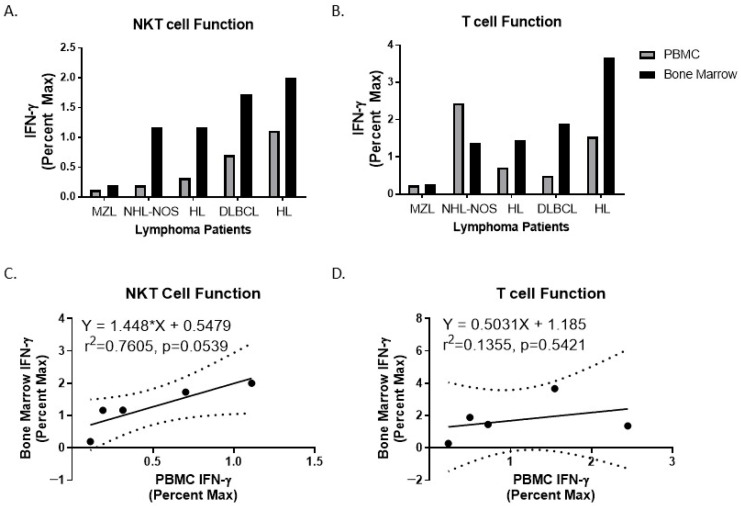
Correlation between NKT and T cell Function in PBMCs and Bone Marrow. PBMCs or Bone Marrow were stimulated with CD1D-Ig/aCD28 aAPC loaded with α-GalCer to activate NKT cells (**A**,**C**) or anti-CD3/CD28 microbeads to stimulate T cells (**B**,**D**) for 4 h. RNA was extracted and qPCR was performed to assess IFN-γ and 18S. Fold induction was calculated relative to the control (cells stimulated with unloaded CD1D-Ig/aCD28 aAPC). Each sample was then compared to its maximum (stimulation with PMA/Ionomycin) to obtain the percent max. Statistical analyses were performed as indicated: (**C**) A linear regression was performed between the NKT function from bone marrow versus PBMC. The assumptions for linearity were evaluated by a Runs test (*p* = 0.5), and a Shapiro–Wilk test for normality demonstrates the residuals were normal (*p* = 0.7048). Thus, a regression equation was used, with the 95% confidence bands plotted as dashed lines. The r^2^ value is indicated, and a F test was used to determine significance, which is shown above. (**D**) A linear regression was performed for the T cell function from bone marrow versus PBMC. The assumptions for linearity were evaluated by a Runs test (*p* > 0.9999), and a Shapiro–Wilk test for normality demonstrates the residuals were normal (*p* = 0.5380). Thus, a regression equation was used, with the 95% confidence bands plotted as dashed lines. The r^2^ value is indicated, and a F test was used to determine significance, which is shown above.

As previously reported, NKT cell number and function are often decreased in cancer patients; however, studies by Molling and colleagues found this decrease in percentage was not observed in IFN-γ-producing NKT cells as determined by ELISPOT, but there was a reduction in absolute number of functional NKT cells in the blood [37]. It was reported that in B cell non-Hodgkin’s lymphoma patients, the percentages of NKT cells were higher and regulatory T cells were lower compared with patients with subsequent disease progression, both pre and post-treatment [38]. Therefore, we sought to determine if NKT cell function, via induction of IFN-γ mRNA upon stimulation, prior to treatment could predict which patients would relapse and which patients would not. While we observed a trend, NKT cell percentages are extremely low in the blood. There have been significant advances in the use of stem cell-derived CAR-NKT cells and in vivo CARs; thus, we asked if immune cell function in the blood correlated with bone marrow-derived immune cells. We found that both NKT cell and T cell function in the peripheral blood positively correlated with NKT cell and T cell function in the bone marrow (Figure 7A,B, *n* = 5), often a tumor site in lymphoma patients. It is worth noting that NKT cell function showed a stronger positive correlation between PBMCs and bone marrow (Figure 7C, *p* = 0.0539) compared to T cell function (Figure 7D, *p* = 0.5421), which further supports the utility of NKT cell function, rather than general T cell function, as a prognostic factor for lymphoma patients.

## 4. Discussion

In this study, we observed a marked reduction in NKT cell functional responsiveness in lymphoma patients relative to healthy donors, with NKT cell activation detected in 82% of healthy donors compared to only 44% of lymphoma patients, indicating substantial impairment of NKT cell activity in the lymphoma setting.

Consistent with systemic immune dysregulation, lymphoma patients exhibited significantly elevated circulating levels of both pro- and anti-inflammatory mediators, including IL-6, IL-10, and Sema4D, compared with healthy controls. Importantly, NKT cell function in peripheral blood positively correlated with NKT cell function in the bone marrow, suggesting that peripheral measurements may serve as a surrogate indicator of bone marrow immune competence in lymphoma patients.

Outcome-associated immune profiling revealed that lower mRNA expression levels of TGF-β, IL-6, IL-10, and IFN-γ were associated with poorer clinical outcomes, whereas higher expression of Sema4D correlated with worse prognosis, highlighting a potentially distinct immunoregulatory role for Sema4D in lymphoma progression.

Together, these findings demonstrate that B-cell lymphoma is associated with impaired NKT cell function and systemic cytokine dysregulation, and they identify Sema4D and NKT cell functional status as candidate immune biomarkers linked to disease outcome.

A limitation of this study is that immune function in lymphoma patients may be influenced by factors beyond relapse risk alone. Treatment heterogeneity, including prior chemotherapy, immunotherapy, and corticosteroid exposure, as well as differences in disease stage, tumor burden, bone marrow involvement, and baseline immune competence, may all contribute to altered NKT cell responses. These variables could confound the relationship between immune dysfunction and clinical outcome and should therefore be addressed in larger, clinically annotated validation cohorts. Future studies incorporating stratified analyses and multivariable modeling will be important for determining whether impaired NKT cell function is independently associated with relapse risk.

From a translational perspective, our qPCR-based NKT cell functional assay has several features that support clinical feasibility, including the use of peripheral blood and reliance on a widely accessible platform, specifically qPCR. Nonetheless, integration into routine practice will require development of a standardized workflow. We have shown the utility of this platform in the context of assessing NKT cell responses in breast cancer patients [15,31]. With further validation, this approach may be most useful as an adjunct immune-monitoring tool to support risk stratification and biomarker-guided clinical decision making in patients with hematological malignancies and solid tumors.

The mechanisms underlying relapse in some patients are not fully understood. Therefore, the ability to predict which patients are more likely to relapse is beneficial. Predictive factors for relapse help physicians focus their time and resources for follow-up and alternate treatment regimens on those who are most likely to need further intervention. To date, this is the first study to our knowledge that identifies NKT cell function as a predictive factor for relapse in B cell lymphoma patients.

NKT cell targeted immunotherapy is not a new field, and the fact that NKT cells can recognize and kill tumor cells is well established. In fact, several studies have found that boosting NKT cell number and function using α-GalCer-loaded DCs as a mechanism of cancer immunotherapy in mice and humans results in increases in NKT cell number correlating with improved outcomes [39,40]. While exciting, it is not surprising that NKT cell number and function were elevated in patients that responded well to α-GalCer-loaded DC therapy, since the treatment was designed to specifically target NKT cells. Interestingly, even in a study that focused on the immune state of lymphoma patients before and after R-CHOP/R-CVP, an immunotherapy that is not specifically designed to target NKT cells, it was found that NKT cell frequencies were increased after therapy in patients that responded relative to patients with subsequent disease progression [40]. However, all these studies relied on characterization of NKT cell number and function after treatment and thus were not able to identify any true predictive factors in their patients.

In this study, we utilized the novel and rapid aAPC-qPCR method, which uses IFN-γ mRNA levels as a readout of NKT cell function, to assess NKT cell function in 50 lymphoma patients representing a range of lymphoma subtypes prior to the initiation of therapy [40]. In addition, we assessed the plasma levels of IL-10, IL-6, Sema4D, and TGF-β1. These patients then went on to receive a variety of treatment regimens and were followed, when possible, to determine their treatment outcome. We identified plasma TGF-β1 and NKT cell function as two factors that significantly predicted which patients would relapse and which patients would not. The significance of both markers (NKT *p* = 0.0404, TGF-β1 *p* < 0.001) is comparable to other markers that have been previously studied, such as BCL-2 (5-year event-free survival *p* = 0.17), BCL-6 (5-year event-free survival *p* = 0.013), Cyclin-D2 (5-year event-free survival *p* < 0.001), IPI score (5-year event-free survival *p* < 0.001), and ESR (2-year event-free survival *p* < 0.001) [41,42].

The role of TGF-β1 in tumor development is complex, with previous studies identifying both pro- and anti-tumorigenic effects [43]. The anti-tumorigenic effects of TGF-β1 were highlighted in a study that found that a conditional knockout of the type II TGF-β receptor in the mouse mammary epithelium resulted in pre-malignant hyperplasia and both shortened the time to tumor development and increased the rate of metastases when crossed onto the PyVmT model of metastatic breast cancer [44]. However, in the field of cancer immunotherapy, the immune-suppressive effects of TGF-β1 are considered a mechanism utilized by tumors to inhibit immunosurveillance and thus promote tumor growth. Indeed, the efficacy of an experimental tumor vaccine in the CT26 colon cancer model can be enhanced by the blockade of TGF-β through treatment with anti-TGF-β [45]. Therefore, while we found our results with TGF-β1 to be unexpected, this is likely because we viewed the results through the lens of cancer immunotherapy, and further studies are needed to elucidate why, in this cohort, TGF-β1 appears to inversely correlate with tumor development and progression.

It is worth noting that this study did not aim to identify the mechanisms by which NKT cell function prior to treatment could serve as a predictor of treatment outcomes in lymphoma. Given the ability of NKT cells to recognize and kill tumor cells, one can conclude that having functional NKT cells may lead to better treatment outcomes due to NKT cell-mediated production of inflammatory cytokines and direct killing of lymphoma. However, the impact of NKT cells may be nuanced. In human Acute Lymphoblastic Leukemia (ALL), the expression of CD1d on the surface of tumor cells was associated with adverse outcomes, while in the same study, CD1d+ ALL cells loaded with α-GalCer were efficiently killed by NKT cell lines [46]. A similar situation is present in Chronic Lymphocytic Leukemia, wherein NKT cells seem to contribute to tumor cell killing in a CD1d-dependent manner despite the fact that CD1d expression on tumor cells is associated with adverse outcomes [47,48].

Conventional T cells have also been long known to be involved in tumor surveillance, yet NKT cell function provided more predictive power for relapse in lymphoma than general T cell function. NKT cells are often referred to as “innate-like,” since they possess many properties considered hallmarks of the innate immune system, such as the ability to respond immediately upon stimulation and the expression of stress-sensing NK cell markers, while simultaneously expressing T-cell receptors that recognize the glycolipid antigens presented on CD1d [49]. It is possible that the inclusion of both innate and adaptive properties makes NKT cells better indicators of the health of the entire immune system, but why and how this would be the case should be the focus of future studies.

## 5. Conclusions

Ultimately, NKT cell function in the peripheral blood, as determined by aAPC-qPCR assessment, can serve as a predictive indicator in lymphoma patients, with higher levels of NKT cell function correlating with a lack of relapse after traditional cytotoxic chemotherapy and thus improved outcomes. This study justifies the implementation of the aAPC-qPCR system in larger and more diverse cohorts of patients to determine the extent of its predictive power. Given its ease of use and ability to quickly provide a result, this system has the promise to assist physicians in implementing care effectively and efficiently.

## 6. Patents

The aAPC technology is patented (US11807675B2; EP3052085B1).

## Figures and Tables

**Table 1:** Study demographics and patient characteristics according to NKT/T cell function ^1^.

**Healthy Donors**			
Characteristic	Number of Patients	NKT Cell Function IFN-γ Fold Change	T Cell Function IFN-γ Fold Change
Total	34	4.968 ± 1.39	22.540 ± 13.07
Age (years)	27/34		
20–39	13	2.195 ± 0.45	9.706 ± 4.02
40–59	11	9.168 ± 4.00	7.289 ± 3.321.90
60+	3	13.167 ± 8.42	13.413 ± 7.35
Sex	34/34		
Female	18	2.541 ± 0.60	36.507 ± 5.07
Male	16	7.698 ± 2.81	24.561 ± 3.32
Race	27/34		
Black	7	3.335 ± 0.75	13.083 ± 3.49
Hispanic	1		
Asian	0		
White	19	7.747 ± 3.72	7.775 ± 3.22
**Lymphoma Patients**			
Characteristic	Number of Patients	NKT Cell Function IFN-γ Fold Change	T Cell Function IFN-γ Fold Change
Total	50	3.720 ± 1.02	5.136 ± 1.61
Age (years)	49/50		
20–39	10	2.336 ± 1.20	1.616 ± 0.35
40–59	19	4.674 ± 2.25	6.262 ± 2.12
60+	20	2.94 ± 1.14	5.958 ± 3.49
Sex	49/50		
Female	21	2.678 ± 1.06	2.007 ± 0.42
Male	28	4.10 ± 1.59	7.577 ± 2.79
Race	49/50		
Black	10	2.696 ± 1.15	2.819 ± 1.01
Hispanic	3	1.170 ± 0.34	24.760 ± 23.32
Asian	2	1.250 ± 0.75	3.600 ± 0.40
White	34	4.060 ± 1.41	4.254 ± 1.25
Pathology	50/50		
HL	12	3.247 ± 1.18	4.630 ± 2.34
DLBCL	10	4.803 ± 2.36	9.310 ± 6.97
FL	9	1.974 ± 0.93	2.628 ± 0.88
MCL	2	1.200 ± 0.30	4.600 ± 1.50
MZL	4	7.925 ± 7.52	8.813 ± 7.71
NHL-NOS	13	3.625 ± 2.51	3.079 ± 0.95
Outcome	44:50		
Relapse	18	1.636 ± 0.61	2.132 ± 0.6244
No Relapse	26	5.78 ± 1.83	4.887 ± 1.57

^1^ Available demographics and immune function scores for all individuals included in this study are highlighted in the table. Ratios adjacent to each characteristic indicate how many individuals had that information available. HL: Hodgkin’s lymphoma; NHL-NOS: non-Hodgkin’s lymphoma—not otherwise specified; DLBCL: diffuse large B cell lymphoma; FL: follicular lymphoma; MCL: mantle cell lymphoma; MZL: marginal zone lymphoma.

## Data Availability

The original contributions presented in this study are included in the article/Supplementary Material. Further inquiries can be directed to the corresponding author.

## Supplementary Materials

The following supporting information can be downloaded at: https://www.mdpi.com/article/10.3390/biom16050749/s1, Figure S1: NKT and T cell function in lymphoma patients can be assessed using CD1d-based aAPC in combination with qPCR. PBMC from lymphoma patients were incubated for 4 h with Media, PMA/ionomycin, anti-CD3/CD28 microbeads, or CD1d-aAPC. NKT cell activation was assessed by measuring IFN-γ mRNA levels by qPCR. Representative data are shown. Median with 95% confidence intervals is reported; Figure S2: Percentage of NKT cells in different lymphoma subtypes. Peripheral blood mononuclear cells (PBMC) were isolated from healthy donors and cancer patients. Cells were stained for Va24+Vb11+ or iNKT+CD3+ and analyzed by flow cytometry. Scatterplots demonstrate the variation in the percentages of NKT cells overall (A), in diffuse large B cell lymphoma (B), follicular lymphoma (C), mantle zone lymphoma (D), non-Hodgkin’s lymphoma not otherwise specified (E), and Hodgkin’s lymphoma (F) patients who did or did not relapse. Median with interquartile range was reported; Figure S3: PBMC were stimulated with CD1D-Ig/aCD28 aAPC loaded with α-GalCer to activate NKT cells or anti-CD3/CD28 microbeads to stimulate T cells for 4 h. RNA was extracted and qPCR was performed to assess IFN-g and 18S. Fold induction was calculated relative to the control (cells stimulated with empty beads). (A–E) NKT cell and (F–J) T cell function in diffuse large B cell lymphoma, follicular lymphoma, mantle zone lymphoma, non-Hodgkin’s lymphoma not otherwise specified, and Hodgkin’s lymphoma patients who did or did not relapse. Median with interquartile range was reported.
