# Supplementary material for: Natural Killer T Cell Function in Lymphoma Patients
Source: Biomolecules. 2026 May 20;16(5):749. doi: 10.3390/biom16050749 (PMC13204881; doi:10.3390/biom16050749)
Supplement: Supplementary file 1 [file biomolecules-16-00749-s001.zip › 052026 Supplemental Figures.pdf]

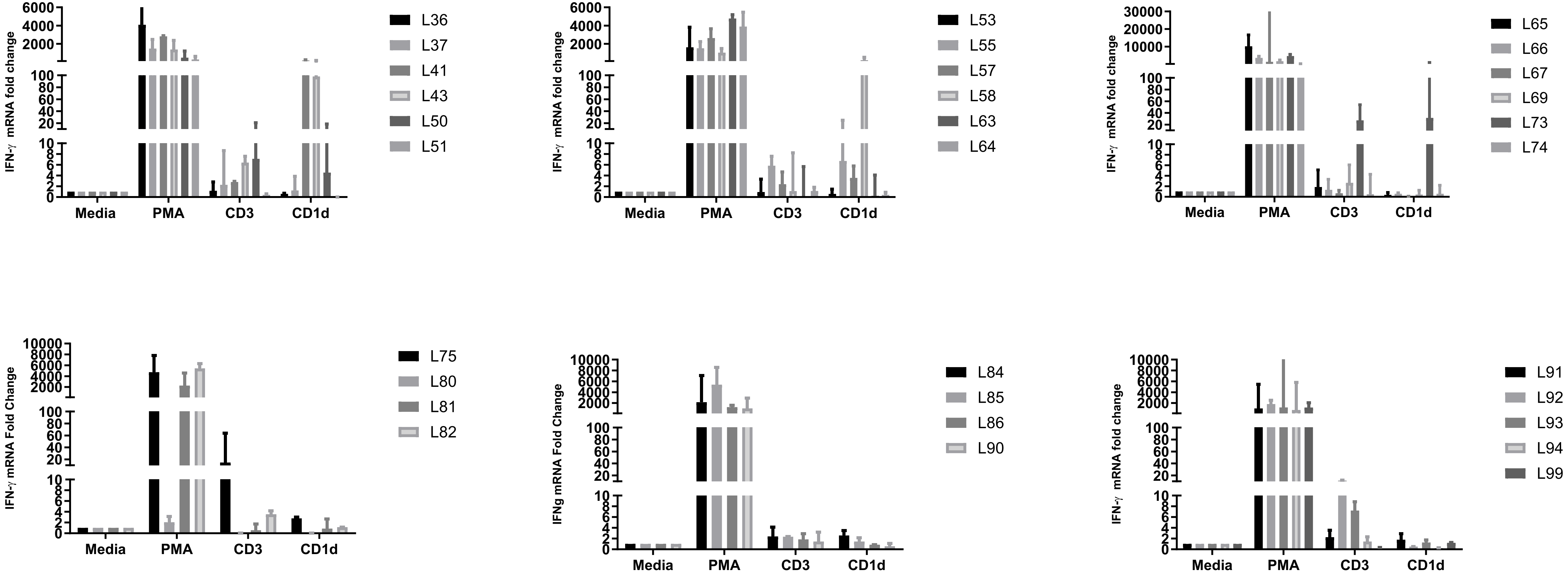

Figure S1. NKT and T cell function in lymphoma patients can be assessed using CD1d-based aAPC in combination with qPCR. PBMC from lymphoma patients were incubated for 4 h with Media, PMA/ionomycin, anti-CD3/CD28 microbeads, or CD1d-aAPC. NKT cell activation was assessed by measuring IFN-γ mRNA levels by qPCR. Representative data are shown. Median with 95% confidence intervals is reported.

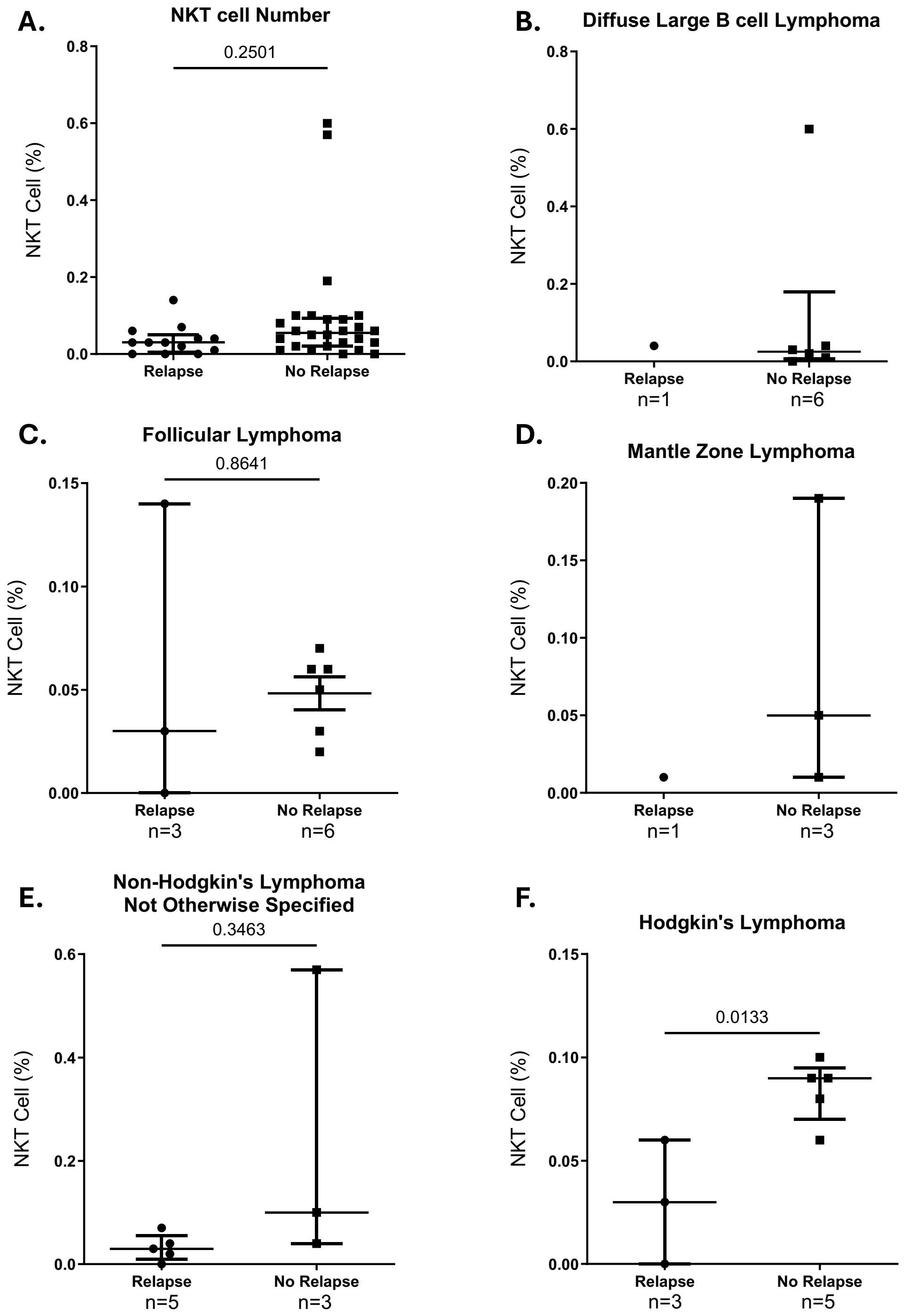

Figure S2. Percentage of NKT cells in different lymphoma subtypes. Peripheral blood mononuclear cells (PBMC) were isolated from healthy donors and cancer patients. Cells were stained for Vα24+Vβ11+ or iNKT+CD3+ and analyzed by flow cytometry. Scatterplots demonstrate the variation in the percentages of NKT cells overall (A), in diffuse large B cell lymphoma (B), follicular lymphoma (C), mantle zone lymphoma (D), non-Hodgkin's lymphoma not otherwise specified (E), and Hodgkin's lymphoma (F) patients who did or did not relapse. Median with interquartile range was reported.

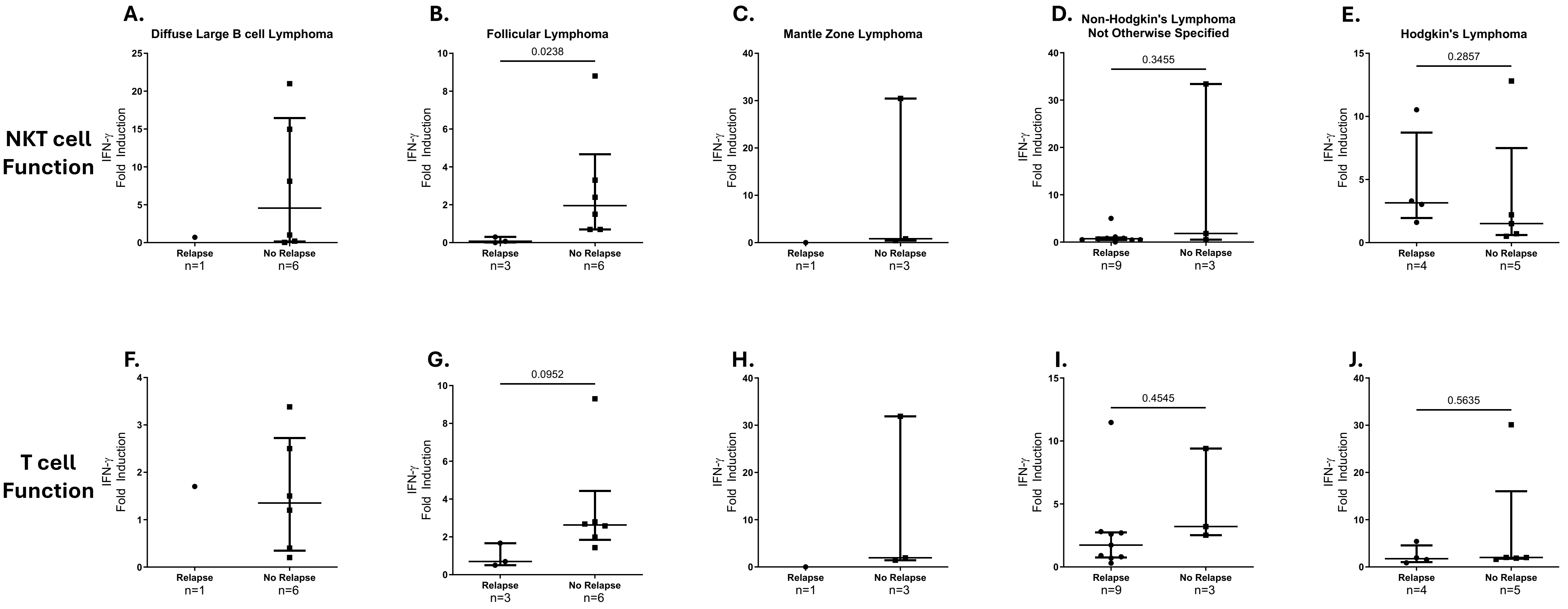

Figure S3. NKT and T cell function in different lymphoma subtypes. PBMC were stimulated with CD1D-Ig/αCD28 aAPC loaded with α-GalCer to activate NKT cells or anti-CD3/CD28 microbeads to stimulate T cells for 4h. RNA was extracted and qPCR was performed to assess IFN-γ and 18S. Fold induction was calculated relative to the control (cells stimulated with empty beads). (A-E) NKT cell and (F-J) T cell function in diffuse large B cell lymphoma, follicular lymphoma, mantle zone lymphoma, non-Hodgkin's lymphoma not otherwise specified, and Hodgkin's lymphoma patients who did or did not relapse. Median with interquartile range was reported.
